# Serotonin concentration enhancers at clinically relevant doses reduce [^11^C]AZ10419369 binding to the 5-HT_1B_ receptors in the nonhuman primate brain

**DOI:** 10.1038/s41398-018-0178-7

**Published:** 2018-07-16

**Authors:** Kai-Chun Yang, Akihiro Takano, Christer Halldin, Lars Farde, Sjoerd J. Finnema

**Affiliations:** 1 Department of Clinical Neuroscience, Center for Psychiatric Research, Karolinska Institutet, Karolinska University Hospital, Stockholm, Sweden; 2 Personalized Health Care and Biomarkers, AstraZeneca PET Science Center at Karolinska Institutet, Stockholm, Sweden; 3 Department of Radiology and Biomedical Imaging, Yale University, New Haven, CT USA

## Abstract

The serotonin (5-HT) system plays an important role in the pathophysiology and treatment of several major psychiatric disorders. Currently, no suitable positron emission tomography (PET) imaging paradigm is available to assess 5-HT release in the living human brain. [^11^C]AZ10419369 binds to 5-HT_1B_ receptors and is one of the most 5-HT-sensitive radioligands available. This study applied 5-HT concentration enhancers which can be safely studied in humans, and examined their effect on [^11^C]AZ10419369 binding at clinically relevant doses, including amphetamine (1 mg/kg), 3,4-methylenedioxymethamphetamine (MDMA; 1 mg/kg) or 5-hydroxy-L-tryptophan (5-HTP; 5 mg/kg). Twenty-six PET measurements (14 for amphetamine, 6 for MDMA and 6 for 5-HTP) using a bolus and constant infusion protocol were performed in four cynomolgus monkeys before or after drug administration. Binding potential (*BP*_ND_) values were determined with the equilibrium method (integral interval: 63–123 min) using cerebellum as the reference region. *BP*_ND_ values were significantly decreased in several examined brain regions after administration of amphetamine (range: 19–31%), MDMA (16–25%) or 5-HTP (13–31%). Reductions in [^11^C]AZ10419369 binding were greater in striatum than cortical regions after administration of 5-HTP, while no prominent regional differences were found for amphetamine and MDMA. In conclusion, [^11^C]AZ10419369 binding is sensitive to changes in 5-HT concentration induced by amphetamine, MDMA or 5-HTP. The robust changes in *BP*_ND_, following pretreatment drugs administered at clinically relevant doses, indicate that the applied PET imaging paradigms hold promise to be successfully used in future human studies.

## Introduction

The serotonin (5-HT) system plays an important role in the pathophysiology and treatment of major psychiatric disorders^1,2^. One approach to examine the functional role of 5-HT in the human brain is to compare outcome measures, such as imaging parameters, cognitive assessment scores or behavior data, before and after manipulation of the endogenous 5-HT concentration^2–5^. Such studies have however been limited due to the lack of non-invasive tools that are sensitive to alterations in 5-HT concentration in the living human brain^3,6^.

Positron emission tomography (PET) is a non-invasive imaging modality which allows examination of changes in endogenous neurotransmitter concentration^4,7,8^. Data are typically interpreted according to the competition model which postulates that the binding of a radioligand to a neuroreceptor will decrease after an increase in neurotransmitter concentration, and vice versa. An alternative interpretation of reductions in radioligand binding is internalization of neuroreceptors following increase in neurotransmitter concentration^8,9^. Several PET radioligands have been reported to be sensitive to drug-induced increases in 5-HT concentration in the nonhuman primate (NHP) brain, including the 5-HT_1A_ receptor radioligand [^11^C]CUMI-101^10^, the 5-HT_1B_ receptor radioligands [^11^C]AZ10419369^11–13^ and [^11^C]P943^14,15^ as well as the 5-HT_2A_ receptor radioligand [^11^C]Cimbi-36^16^. Of hitherto developed radioligands, [^11^C]AZ10419369, a partial agonist for the 5-HT_1B_ receptor, is one of the most sensitive. [^11^C]AZ10419369 binding has been demonstrated to be sensitive to increases in 5-HT concentration induced by fenfluramine 5 mg/kg (~50% binding reductions)^12^ or escitalopram 2 mg/kg (~10% binding reductions)^13^ in the cortex of the monkey brain. Moreover, serotonergic drug-induced decreases in [^11^C]AZ10419369 binding have recently been associated with increases in extracellular 5-HT concentration measured by microdialysis in the pig brain^17^. Therefore, [^11^C]AZ10419369 is a promising radioligand to evaluate increases in 5-HT concentration in the human brain.

However, the translation of these PET imaging paradigms to clinical 5-HT research has been limited. Fenfluramine has provided the largest reductions in radioligand binding in NHP^11,12^ but has been applied in only one human PET study^18^ since it has been removed from the market because of cardiovascular toxicity^19,20^. Clinically relevant doses of selective serotonin reuptake inhibitor (SSRI) have also not provided a method suitable for measurement of acute 5-HT release capacity^13,21,22^. In summary, although suitable PET radioligands have been available, a pharmacological challenge suitable to examine increases in endogenous 5-HT concentration in humans is still warranted.

There are a variety of 5-HT concentration elevating compounds, with diverse mechanisms of action^8,23,24^. Here, microdialysis studies can provide guidance in the selection of suitable experimental drugs, as changes in radioligand binding likely relate to magnitude of elevation in 5-HT concentration in extracellular fluid^7,8^. The 5-HT releaser fenfluramine has been reported to induce a 20-fold increase in peak 5-HT concentration in the monkey brain at the dose of 5 mg/kg^25^. For other 5-HT releasers, similar robust 5-HT release has been reported in the rodent brain, such as amphetamine (~6-fold peak increase at 2.5 mg/kg)^26^ and 3,4-methylenedioxymethamphetamine (MDMA) (~20-fold peak increase at 5 mg/kg)^27^. In addition, 20 mg/kg of 5-hydroxy-L-tryptophan (5-HTP), the precursor to 5-HT, has been shown to cause an about 200-fold increase in peak 5-HT concentration in the monkey brain^28^. Interestingly, these alterations are larger than the twofold to fivefold increase in 5-HT concentration induced by very high doses of SSRI in the rodent brain^29^. Therefore, amphetamine, MDMA and 5-HTP have the potential to produce detectable increases in 5-HT concentration and to be utilized in human studies.

The aim of the current study was to evaluate the effect of pretreatment with three 5-HT concentration enhancers on [^11^C]AZ10419369 binding in the NHP brain. Amphetamine was selected for its high translational potential supported by the established use in numerous human imaging studies^4,30–32^. MDMA was selected based on its larger release of 5-HT than dopamine^27,33^. 5-HTP was selected based on a more selective increase in 5-HT concentration when compared with 5-HT releasers^34^ and a different underlying mechanism^24^. To enable translation into future human studies, the selected doses were a trade-off between robustness of increases in 5-HT concentration and applicability in human studies. We hypothesized that [^11^C]AZ10419369 binding would decrease following administration of any of these three drugs.

## Materials and methods

### Subjects

The NHP study was approved by the Animal Research Ethical Committee of the Northern Stockholm region (Dnr N386/09, N452/11, N632/12, N633/12 and N185/14), and four female cynomolgus monkeys (*Macaca fascicularis*) with body weight of 4.2 ± 1.2 kg (mean ± SD) were included (Table 1). No randomization or blinding for NHP allocation was applied. The sample size was selected based on the reported effect of escitalopram on [^11^C]AZ10419369 binding (see Supplementary Methods). The caring and experimental procedures were performed according to the ‘Guidelines for planning, conducting and documenting experimental research’ (Dnr 4820/06-600) of Karolinska Institutet and the ‘Guide for the Care and Use of Laboratory Animals: Eighth Edition’^35^.

**Table 1:** Study details including NHP characteristics and number of PET measurements in which each NHP participated

	NHP1	NHP2	NHP3	NHP4
Age (years old)^a^	5	5	7	11
Body weight (kg)^a^	3.7	4.5	3.7	7.3
*D*-amphetamine^b^	6	4	4	
3,4-methylenedioxymethamphetamine (MDMA)^b^	2	2	2	
5-hydroxy-L-tryptophan (5-HTP)^b^	2	2		2

^a^Mean of the values across all PET measurements

^b^Note that half of the number of PET measurements reflect baseline conditions and half post-drug conditions

### Preparation of [^11^C]AZ10419369

[^11^C]AZ10419369 was prepared according to the procedures reported previously^36^.

### Study design

**Fig. 1:**
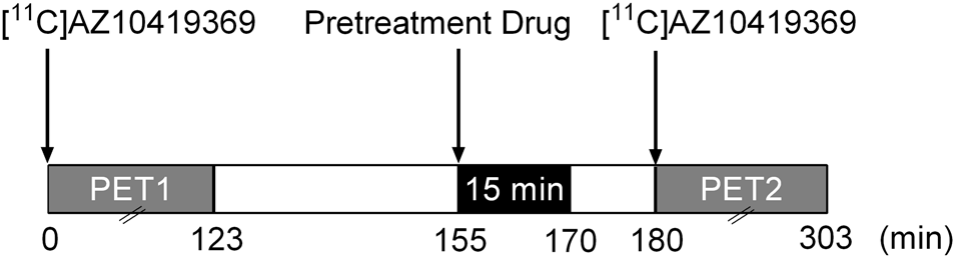
Schematic summary of an experimental day, including two PET measurements with the radioligand [^11^C]AZ10419369 and intravenous infusion of a pretreatment drug in a nonhuman primate (NHP). PET positron emission tomography

A total of 26 PET measurements with a bolus/infusion (B/I) protocol were performed on 13 experimental days (*n* = 14 for amphetamine in 3 NHPs and *n* = 6 for MDMA and 5-HTP in 3 NHPs). The experimental conditions of each NHP are summarized in Table 1. The interval between experimental days in the same NHP was at least 1 month to minimize confounding carry-over effects. On each experimental day, a baseline PET measurement was performed in the morning followed by a second PET measurement in the afternoon after pretreatment with any of the three experimental drugs. The two PET measurements were performed approximately 3 h apart (Fig. 1).

### Drug administration

All pretreatment drugs were formulated in phosphate-buffered saline and the reported doses refer to the salt when applicable. *D*-amphetamine (1 mg/kg, sulfate salt), MDMA (1 mg/kg, hydrochloride salt) or 5-HTP solution (5 mg/kg) was infused intravenously (~1 mlL/kg) over 15 min, starting approximately 25 min prior to the injection of [^11^C]AZ10419369 (Fig. 1).

### PET experimental procedures

Anesthesia was initiated by intramuscular injection of ketamine hydrochloride (~10 mg/kg) and maintained by a mixture of sevoflurane (2–8%), oxygen and medical air. PET measurements were conducted in the high-resolution research tomograph (HRRT)^37^. A 6 min transmission measurement (using a single Cesium-137 source) was followed by the acquisition of list-mode data for 123 min after injection of [^11^C]AZ10419369. [^11^C]AZ10419369 was administered intravenously using a B/I protocol with the bolus-to-infusion rate ratio (*K*_bol_) ranging from 80 to 180 min. This B/I protocol has been validated in previous NHP studies^12,13^.

### Magnetic resonance imaging (MRI)

T1-weighted MRI images were acquired for each monkey on a 1.5 Tesla Signa MRI scanner (General Electronics, Milwaukee, WI) using a three-dimensional (3D) spoiled gradient recalled (SPGR) protocol with repetition time 21 ms, flip angle 35°, field of view 12.8, matrix 256 × 256 × 128, 128 × 1.0 mm^2^ slices.

### Image data analysis and quantification

The MRI images were reoriented to the anterior–posterior commissure plane and non-brain tissues were removed manually using the Image Processing and VOI Analysis Tool (PBAS) in PMOD (version 3.704; PMOD Technologies, Zurich, Switzerland). The processed brain MRI images were then corrected for inhomogeneous intensity by applying the N4 algorithm^38^ using the Advanced Normalization Tools (ANTs) software package (http://stnava.github.io/ANTs/) (see Supplementary Methods). PET images were preprocessed according to previously reported methods^37^ with reconstructed image frames binned as: 3 × 60 s, 6 × 180 s and 17 × 360 s.

Each subject’s baseline summed PET image (average of time frames corresponding to 0–57 min) was coregistered to its individual MRI brain image by the Rigid matching algorithm in the PMOD Fuse It Tool (PFUSEIT). The resulting transformation matrices were applied to the two corresponding PET measurements performed on the same day.

Volumes of interest (VOIs) were defined on an in-house cynomolgus brain template that has been generated from MRI images of 36 cynomolgus monkeys using symmetric group-wise normalization (SyGN) procedures^39^ provided by ANTs. Except for whole brain (WB), the automated delineation of VOIs was guided by the NeuroMaps atlas in the INIA19 template^40^ and the Paxinos’ histology atlas^41^ in the CIVM template^42^ (see Supplementary Methods). Each individual brain MRI image was normalized to the in-house cynomolgus brain template by the antsRegistration algorithm using symmetric image normalization method (SyN)^43^ in ANTs. The resulting normalization matrix was used to inversely transform the template VOIs into the individual MRI space. The WB VOI was manually defined on individual MRI image.

Based on the known 5-HT_1B_ receptor distribution, 11 VOIs were included in the current study, including 2 neocortical regions: frontal cortex (FC) and occipital cortex (OC); 4 striatal regions: putamen, caudate nucleus (CN), ventral striatum (VS) and globus pallidus (GP); 1 limbic region: hippocampus; and finally VOIs for thalamus, midbrain, cerebellum and WB.

### Calculation of binding potential and related outcome measures

For each VOI, a decay-corrected time–activity curve was generated from the coregistrated dynamic PET data. Binding potential (*BP*_ND_) values were calculated using the equilibrium method (EM; integral interval: 63 to 123 min) with cerebellum as the reference region^12,44,45^. Parametric *BP*_ND_ images were generated with EM and normalized to the cynomolgus brain template.

The relative change in *BP*_ND_ values (∆*BP*_ND_) (%) was calculated using the following equation:

1 $$\Delta BP_{\mathrm {ND}}\left( \% \right)\, = \,\frac{{(BP_{{\mathrm{ND}}}{\mathrm{Pretreatment}} - BP_{{\mathrm{ND}}}{\mathrm{Baseline}})}}{{BP_{{\mathrm{ND}}}{\mathrm{Baseline}}}}{\mathrm{ \times 100}}.$$

### Statistical analysis

A paired *t*-test was used to assess changes in parameters between the two PET measurements performed on the same day. For the amphetamine studies which were repeated in the same NHP, the mean value of all baseline and pretreatment PET measurements for each NHP were used to represent the baseline and pretreatment PET measurements of NHP, respectively. All statistical analyses were performed in GraphPad Prism (version 6.05; GraphPad Software Inc., La Jolla, CA, USA). The threshold of significance was set as *P* *<* 0.05 (two-tailed). Considering the small sample size and the exploratory nature of the current study, no correction for multiple comparisons was performed.

## Results

### Radiochemistry

The radiochemical purity of [^11^C]AZ10419369 was above 97% (*n* = 26). There were no significant differences in injected radioactivity, specific radioactivity or injected mass between baseline and pretreatment PET measurements (Table S1).

### Changes in [^11^C]AZ10419369 *BP*_ND_ values following drug administration

**Fig. 2:**
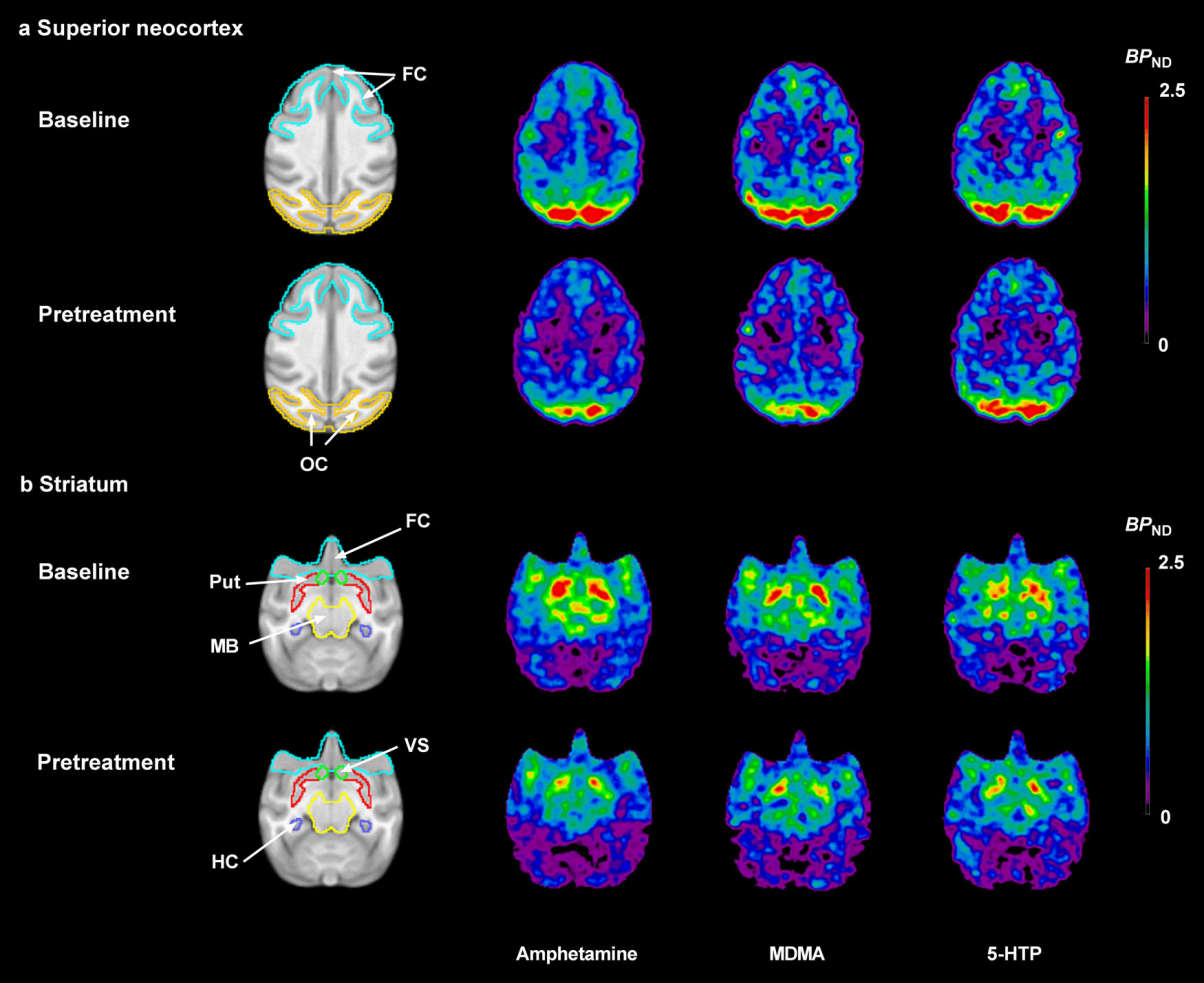
Mean parametric *BP*_ND_ images of [^11^C]AZ10419369 derived by the equilibrium method at baseline (different *n* for each pretreatment condition) and following pretreatment with 3 different experimental drugs (*n* = 3 for each experimental condition): amphetamine 1 mg/kg, 3,4-methylenedioxymethamphetamine (MDMA) 1 mg/kg and 5-hydroxy-L-tryptophan (5-HTP) 5 mg/kg. The images were normalized to an in-house cynomolgus brain template. **a** Axial view of images at the level of superior neocortex. **b** Axial view of images at the level of striatum. FC frontal cortex, HC hippocampus, MB midbrain, OC occipital cortex, Put putamen, VS ventral striatum

**Fig. 3:**
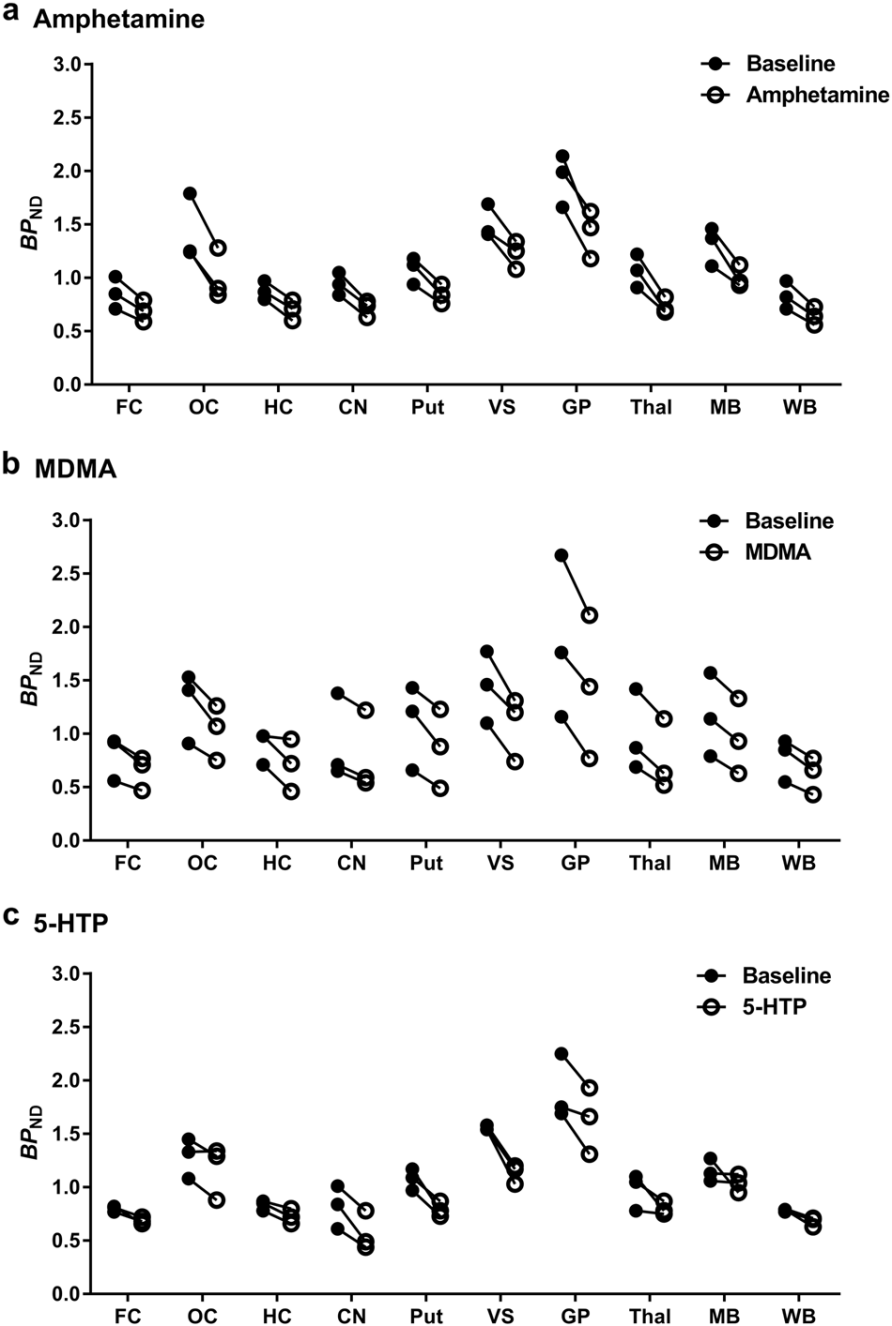
Individual *BP*_ND_ values before and after pretreatment with three different pretreatment drugs in 10 predefined volumes of interest (*n* = 3 for each experimental condition). **a** Amphetamine 1 mg/kg. **b** 3,4-Methylenedioxymethamphetamine (MDMA) 1 mg/kg. **c** 5-Hydroxy-L-tryptophan (5-HTP) 5 mg/kg. CN caudate nucleus, FC frontal cortex, GP globus pallidus, HC hippocampus, MB midbrain, OC occipital cortex, Put putamen, Thal thalamus, VS ventral striatum, WB whole brain

Following administration of pretreatment drugs, the radioactivity concentration was reduced in several VOIs when compared with baseline (Fig. 2). The regional changes in *BP*_ND_ values (EM) after pretreatment are given in Table 2 and Fig. 3. For the WB, the mean reduction in *BP*_ND_ for amphetamine, MDMA and 5-HTP were 23%, 20% and 14%, respectively. Amphetamine decreased *BP*_ND_ values significantly in all examined brain regions (range: 19–31%). The regional *BP*_ND_ values of each baseline and pretreatment PET measurement in the amphetamine experiments are presented in Table S2. For MDMA, the reduction in [^11^C]AZ10419369 *BP*_ND_ was statistically significant in all examined brain regions, except hippocampus. In contrast, 5-HTP only significantly reduced *BP*_ND_ in 5 brain regions: FC, hippocampus, CN, putamen and VS (range: 13–31%). After administration of 5-HTP, the *BP*_ND_ reductions in striatum (28%) were prominently larger than those in FC (14%), while the *BP*_ND_ reductions of the two 5-HT releasers were comparable between striatum (21%) and FC (19%).

**Table 2:** Effect of pretreatment drugs on regional binding potential (*BP*_ND_) values

Region	Amphetamine 1.0 mg/kg (*n* = 3)^a^			MDMA 1.0 mg/kg (*n* = 3)			5-HTP 5.0 mg/kg (*n* = 3)		
	Baseline	PreTx	∆*BP*_ND_ (%)	Baseline	PreTx	∆*BP*_ND_ (%)	Baseline	PreTx	∆*BP*_ND_ (%)
Frontal cortex	0.86	0.69	−19*	0.81	0.65	−19*	0.80	0.68	−14*
Occipital cortex	1.43	1.01	−30*	1.28	1.03	−20*	1.29	1.17	−10
Hippocampus	0.88	0.70	−20**	0.89	0.71	−22	0.83	0.73	−13*
Caudate nucleus	0.94	0.72	−24*	0.92	0.78	−16*	0.82	0.57	−31*
Putamen	1.08	0.85	−22*	1.10	0.87	−23*	1.08	0.79	−26*
Ventral striatum	1.51	1.22	−19*	1.44	1.08	−25*	1.55	1.13	−27*
Globus pallidus	1.93	1.42	−26*	1.86	1.44	−24*	1.90	1.63	−14
Thalamus	1.07	0.74	−31*	0.99	0.77	−24*	0.98	0.80	−17
Midbrain	1.32	1.00	−23*	1.17	0.96	−18*	1.15	1.04	−9
Whole brain	0.83	0.64	−23*	0.78	0.62	−20*	0.79	0.68	−14

Data presented as mean values.

*PreTx* pretreatment; ∆*BP*_ND_ (%) = $\frac{{({\it{BP}}_{{\mathrm{ND}}}{\mathrm{Pretreatment}} - {\it{BP}}_{{\mathrm{ND}}}{\mathrm{Baseline}})}}{{{\it{BP}}_{{\mathrm{ND}}}{\mathrm{Baseline}}}}{\mathrm{ \times 100}}$

^a^A total of 14 PET measurements were performed in 3 NHPs (NHP1: 6, NHP2: 4 and NHP3: 4; half for baseline conditions and half for pretreatment conditions). For each NHP, the mean value of all related PET measurements was used to represent the value of the NHP

*P < 0.05

**P < 0.01 (two-tailed) by paired t-test

## Discussion

The aim of the present study was to develop a PET methodology suitable for translation to human studies evaluating changes in endogenous 5-HT concentration following drug challenges. A previously established BI protocol was used to demonstrate that [^11^C]AZ10419369 binding to the 5-HT_1B_ receptor is sensitive to increases in 5-HT concentration induced by amphetamine, MDMA or 5-HTP in the NHP brain. Interestingly, the effect of the 5-HT precursor 5-HTP was larger in striatum than frontal cortex, while there were no prominent regional differences for the 5-HT releasers amphetamine and MDMA. The current results suggest that all three PET imaging paradigms have potential to be utilized in future human studies investigating changes in the endogenous 5-HT concentration.

The pretreatment drugs used in the current study belong to two groups with different modes of action. Amphetamine is a prototypic substrate of monoamine transporters and MDMA is a substituted amphetamine derivative. Both drugs are structurally similar to fenfluramine and function as 5-HT releasers^23,46^. These 5-HT releasers have been suggested to increase endogenous 5-HT concentration by several mechanisms including competitive inhibition of 5-HT uptake, reverse transport of 5-HT, transporter trafficking and inhibition of monoamine oxidase^23,46,47^. Considering the similarity in structure of different monoamines, the releasing effect is not only specific for 5-HT but also includes dopamine and norepinephrine. On the other hand, the 5-HT precursor 5-HTP increases endogenous 5-HT concentration by facilitating 5-HT synthesis and has thereby a limited effect on other monoamines^24,34^. Consequently, the different modes of action between 5-HT releasers and 5-HTP should be considered when interpreting the present results or selecting pretreatment drugs for future studies.

The observed effect of *D*-amphetamine 1 mg/kg on [^11^C]AZ10419369 binding is in agreement with a previous report using [^11^C]P943 in baboon^14^. Amphetamine has been widely applied in PET studies to evaluate dopamine release in human subjects^4,7,30–32^. Interestingly, the reduction in [^11^C]AZ10419369 *BP*_ND_ (18 to 31%) was comparable with that for [^11^C]raclopride *BP*_ND_ (23–44%) reported in previous NHP studies after administration of 1 mg/kg of amphetamine^48,49^. Importantly, the sensitivity of [^11^C]raclopride binding to dopamine release induced by lower dose of amphetamine (e.g., ~16% reduction after 0.3 mg/kg intravenous) has been confirmed in human studies^4,7,50^. Moreover, one recent human study^51^ has reported that 0.5 mg/kg oral dose of *D*-amphetamine reduced the binding of [^11^C]Cimbi-36, a radioligand with a 5-HT sensitivity comparable to [^11^C]AZ10419369^16^. Altogether, it is expected that in the human brain, the current PET imaging paradigm could be sensitive to 5-HT release induced by amphetamine with doses that have been applied in previous human PET studies (~0.5 mg/kg oral or ~0.3 mg/kg intravenous)^52^.

In contrast to amphetamine, MDMA seems to be a slightly more efficient releaser of 5-HT than of dopamine in the rodent brain^27^. MDMA has been shown to elevate 5-HT concentrations to about twofold higher degree than equivalent doses of amphetamine^53^. The dose of MDMA (1 mg/kg) in the present study is comparable with doses in previous human studies (~1.7 mg/kg oral) and is unlikely to give rise to neurotoxicity or significant adverse events^54–57^. In the present study in NHP, the decrease in [^11^C]AZ10419369 binding was similar between 1 mg/kg of MDMA and 1 mg/kg of amphetamine. MDMA may thus serve as an alternative to the use of amphetamine for examination of the responsiveness of the 5-HT system in clinical studies.

The 5-HT precursor 5-HTP (5 mg/kg) induced a 30% decrease in striatal [^11^C]AZ10419369 *BP*_ND_. This marked effect is consistent with a previous report in which 20 mg/kg of 5-HTP reduced [^11^C]DASB binding to the serotonin transporter in the NHP brain (~43% in striatum)^28^. The dose of 5-HTP (5 mg/kg) in the present study was comparable with the clinical dose range of 200 to 300 mg/day, typically administered per os or intravenous^24^. Furthermore, a slow-release (SR) formulation of 5-HTP has been shown to provide more stable increases in 5-HT concentration, with less adverse effects, than the immediate-release form in rodent studies^58^. Therefore, PET measurement with [^11^C]AZ10419369 and pretreatment with oral administration of 5-HTP SR is a promising paradigm to investigate 5-HT release in the human brain.

The observed larger 5-HTP-induced reduction in [^11^C]AZ10419369 binding in striatum than cortical regions is also consistent with previous results with [^11^C]DASB in the monkey brain^28^. This regional effect is in line with microdialysis studies in NHP demonstrating a larger increase (~27-fold) in 5-HT concentration in striatum than in prefrontal cortex induced by 5-HTP^28^. Interestingly, 5-HTP has been labeled with carbon-11 and examined as a PET marker for 5-HT synthesis^59^. The [^11^C]5-HTP influx rate has been shown to be higher in striatum than in prefrontal cortex in the NHP brain^28^. Together, the observed regional effect is likely attributed to the regional differences in the activity of amino acid decarboxylase (AADC) which converts 5-HTP to 5-HT^28^. In summary, the regional difference in the effect of 5-HTP on [^11^C]AZ10419369 binding supports the use of this PET imaging paradigm to detect changes in 5-HT concentration, primarily in subcortical regions.

[^11^C]5-HTP has been applied to examine 5-HT synthesis in patients with different neuropsychiatric disorders. For example, increases in [^11^C]5-HTP influx rate in striatum and cortical regions have been reported in patients with social anxiety disorder^60^, while decreases in several brain regions has been observed in patients with depression^61^. It is worth noting that [^11^C]5-HTP is also trapped in the candidate reference regions (e.g., cerebellum) which significantly biases quantification using reference tissue models. A modified reference Patlak method has been proposed to deal with this problem^62,63^. In this approach, the value of the trapping rate constant in the reference region should be measured or obtained from the literature. This need might limit the utility of [^11^C]5-HTP for clinical studies^59^. Accordingly, the current PET imaging paradigm with pretreatment of 5-HTP might be an alternative way to evaluate 5-HT synthesis in the human brain.

Several factors should be considered when selecting one of these three pretreatment drugs to examine increases in 5-HT concentration in the human brain. First, for amphetamine and MDMA, the significant *BP*_ND_ changes were observed in most brain regions while the effect of 5-HTP was mainly located in striatum which may limit the evaluation of increases in 5-HT concentration in cortical regions. Second, although 5-HTP is specific for increases in 5-HT concentration, amphetamine and MDMA may also release other monoamine neurotransmitters such as dopamine and norepinephrine^26,27,64,65^. Third, in contrast to the strict regulations on amphetamine and MDMA (e.g., Schedule II and Schedule I of the Controlled Substances Act in the United States, respectively), 5-HTP is a commercially available dietary supplement^24^ and may from a regulatory perspective be more easily applied in human PET studies. Accordingly, in future human studies the choice among these three PET imaging paradigms has to be based on the specific study objectives.

To enable the use of these three experimental drugs in clinical studies, it is important to determine the corresponding doses to be administered to humans. One approach is to estimate human equivalent doses by using an allometric scaling approach in which doses are normalized across species based on the body surface area^66^. According the Food and Drug Administration guide^67^, the dose conversion factor from rhesus monkey to human is 0.324. Moreover, considering differences in the route of drug administration between NHP and human (intravenous and oral, respectively), the bioavailability of each experimental drug should also be included in the calculations^68^. The bioavailability of methamphetamine and 5-HTP (co-administration of AADC inhibitor) have been reported as 67%^69^ and 48–69%^70,71^, respectively. The bioavailability of MDMA was not available from the literature. Consequently, the calculated human oral doses are ~0.5 mg/kg for *D*-amphetamine and 2.3–3.4 mg/kg for 5-HTP. Future human PET studies are warranted to evaluate the effect of these experimental drugs at potentially relevant doses.

A limitation of the current study could be that factors other than increases in 5-HT concentration may decrease [^11^C]AZ10419369 binding. First, direct occupancy of pretreatment drugs on 5-HT_1B_ receptor should be considered. Although plasma concentrations of pretreatment drugs were not measured in the current study, estimated direct occupancy of MDMA 75 mg or its main active metabolites, 3,4-methylenedioxyamphetamine (MDA) (both with *K*_i_ > 10,000 nM), on the human 5-HT_1B_ receptor has been reported to be <10% or 0.6%, respectively^56^. Because the affinity of amphetamine and 5-HTP to the 5-HT_1B_ receptor could not be found in the literature, direct occupancy could not be estimated for these two drugs. Second, the influence of release of other neurotransmitters, such as dopamine or norepinephrine, after administration of amphetamine or MDMA on the present results should be considered. Based on data from the National Institute of Mental Health’s Psychoactive Drug Screening Program (NIMH PDSP)^72^, the affinity of dopamine and norepinephrine to the 5-HT_1B_ receptor (both with *K*_i_ > 10,000 nM) is much lower than that of 5-HT (*K*_i_: 2–24 nM). Therefore, the contributions of dopamine and norepinephrine to the reductions in [^11^C]AZ10419369 binding can be assumed to be minimal. In conclusion, a major proportion of the drug-induced decrease in [^11^C]AZ10419369 binding can be attributed to 5-HT release.

Another limitation of the current animal study is the use of anesthesia. Ketamine has been reported to increase extracellular 5-HT concentration and thereby to reduce radioligand binding to the 5-HT transporter in the NHP brain^73,74^. However, ketamine has been shown to increase [^11^C]AZ10419369 binding in several regions in the NHP brain (nucleus accumbens, ventral part of GP and the midline nucleus reuniens of thalamus)^73^. This effect is potentially related to ketamine-induced upregulation of 5-HT_1B_ receptors^73^. Such upregulation of 5-HT_1B_ receptors has been associated with sensitization of psychostimulant effects in rodents^75^. Importantly, in the same NHP [^11^C]AZ10419369 study, Yamanaka et al.^73^ also demonstrated that fenfluramine-induced decreases in [^11^C]AZ10419369 binding were similar in awake and anesthetized monkeys. Therefore, a potential effect of ketamine on 5-HT concentration or 5-HT_1B_ receptor expression is likely not the main cause of the presently observed drug-induced reductions in [^11^C]AZ10419369 binding. Currently, there are no studies reported that evaluate the effect of sevoflurane on [^11^C]AZ10419369 binding. In the current study we minimized the effect of sevoflurane by maintaining constant levels of volatile anesthetics during PET measurements performed on the same experimental day. Future studies evaluating the current PET imaging paradigms in conscious humans are warranted to exclude potential anesthesia effects.

Several other factors also need to be considered when interpreting the current results. First, the sample size is small. Second, only female NHPs were included thereby potentially limiting extrapolation of the results to male subjects. Finally, drug-induced changes in cerebellar distribution volume (*V*_ND_) were not controlled for as no blood sampling was performed. However, since high doses of 5-HT_1B_ receptor antagonists did not decrease cerebellar [^11^C]AZ10419369 binding in previous human studies^76^, we expect the possible effect of experimental drugs on cerebellar [^11^C]AZ10419369 binding to be minimal. In summary, although these potentially confounding factors are present, they are unlikely to change the main results of the current study.

In conclusion, our results suggest that [^11^C]AZ10419369 binding is sensitive to increases in 5-HT concentration induced by amphetamine, MDMA or 5-HTP in the primate brain. Considering the robust *BP*_ND_ changes and the clinically relevant doses of applied pretreatment drugs, it is likely that the current PET imaging paradigms could be successfully translated in future human studies aiming at understanding the function of the 5-HT system in the pathophysiology and treatment of neuropsychiatric disorders.

## Electronic supplementary material

Supplementary information_clear

## References

[1] Chou YH (2012). Impaired cognition in bipolar I disorder: the roles of the serotonin transporter and brain-derived neurotrophic factor. J. Affect. Disord..

[2] Cools R, Roberts AC, Robbins TW (2008). Serotoninergic regulation of emotional and behavioural control processes. Trends Cogn. Sci..

[3] Faulkner P, Deakin JFW (2014). The role of serotonin in reward, punishment and behavioural inhibition in humans: insights from studies with acute tryptophan depletion. Neurosci. Biobehav. Rev..

[4] Finnema SJ (2015). Application of cross-species PET imaging to assess neurotransmitter release in brain. Psychopharmacology.

[5] Rogers RD (2011). The roles of dopamine and serotonin in decision making: evidence from pharmacological experiments in humans. Neuropsychopharmacology.

[6] van Donkelaar EL (2011). Mechanism of acute tryptophan depletion: is it only serotonin?. Mol. Psychiatry.

[7] Laruelle M (2000). Imaging synaptic neurotransmission with in vivo binding competition techniques: a critical review. J. Cereb. Blood Flow Metab..

[8] Paterson LM, Tyacke RJ, Nutt DJ, Knudsen GM (2010). Measuring endogenous 5-HT release by emission tomography: promises and pitfalls. J. Cereb. Blood Flow Metab..

[9] Sibon I (2008). Decreased [18F]MPPF binding potential in the dorsal raphe nucleus after a single oral dose of fluoxetine: a positron-emission tomography study in healthy volunteers. Biol. Psychiatry.

[10] Milak MS (2011). In vivo serotonin-sensitive binding of [11C]CUMI-101: a serotonin 1A receptor agonist positron emission tomography radiotracer. J. Cereb. Blood Flow Metab..

[11] Finnema SJ (2010). Fenfluramine-induced serotonin release decreases [11C]AZ10419369 binding to 5-HT1B-receptors in the primate brain. Synapse.

[12] Finnema SJ, Varrone A, Hwang TJ, Halldin C, Farde L (2012). Confirmation of fenfluramine effect on 5-HT(1B) receptor binding of [(11)C]AZ10419369 using an equilibrium approach. J. Cereb. Blood Flow Metab..

[13] Nord M, Finnema SJ, Halldin C, Farde L (2013). Effect of a single dose of escitalopram on serotonin concentration in the non-human and human primate brain. Int. J. Neuropsychopharmacol..

[14] Ridler K (2011). Characterization of in vivo pharmacological properties and sensitivity to endogenous serotonin of [11C] P943: a positron emission tomography study in Papio anubis. Synapse.

[15] Cosgrove KP (2011). Assessing the sensitivity of [11C]p943, a novel 5-HTIB radioligand, to endogenous serotonin release. Synapse.

[16] Yang KC (2017). Fenfluramine reduces [11C]Cimbi-36 binding to the 5-HT2A receptor in the nonhuman primate brain. Int. J. Neuropsychopharmacol..

[17] Cerebral serotonin release correlates with [11C]AZ10419369 PET measures of 5-HT1B receptor binding inthe pig brain. *J. Cereb. Blood Flow Metab.* 2017; e-pub ahead of print 7 July 2017; 10.1177/0271678X17719390. 10.1177/0271678X17719390 PMC6434452 28685616

[18] Quednow BB (2012). Assessment of serotonin release capacity in the human brain using dexfenfluramine challenge and [18F]altanserin positron emission tomography. NeuroImage.

[19] Hutcheson JD, Setola V, Roth BL, Merryman WD (2011). Serotonin receptors and heart valve disease-It was meant 2B. Pharmacol. Ther..

[20] Montani D, Seferian A, Savale L, Simonneau G, Humbert M (2013). Drug-induced pulmonary arterialhypertension: a recent outbreak. Eur. Respir. Rev..

[21] Pinborg LH (2012). No change in [11C]CUMI-101 binding to 5-HT1A receptors after intravenous citalopram in human. Synapse.

[22] Selvaraj S (2012). Measuring endogenous changes in serotonergic neurotransmission in humans: a [11C]CUMI-101 PET challenge study. Mol. Psychiatry.

[23] Rothman RB, Baumann MH (2002). Therapeutic and adverse actions of serotonin transporter substrates. Pharmacol. Ther..

[24] Turner EH, Loftis JM, Blackwell AD (2006). Serotonin a la carte: supplementation with the serotonin precursor 5-hydroxytryptophan. Pharmacol. Ther..

[25] Udo de Haes JI, Harada N, Elsinga PH, Maguire RP, Tsukada H (2006). Effect of fenfluramine-induced increases in serotonin release on [18F]MPPF binding: a continuous infusion PET study in conscious monkeys. Synapse.

[26] Millan MJ (1999). Contrasting mechanisms of action and sensitivity to antipsychotics of phencyclidine versus amphetamine: importance of nucleus accumbens 5-HT 2A sites for PCP-induced locomotion in the rat. Eur. J. Neurosci..

[27] Gołembiowska K, Jurczak A, Kamińska K, Noworyta-Sokołowska K, Górska A (2016). Effect of some psychoactive drugs used as ‘Legal Highs’ on brain neurotransmitters. Neurotox. Res..

[28] Yamamoto S, Onoe H, Tsukada H, Watanabe Y (2007). Effects of increased endogenous serotonin on the in vivo binding of [11C]DASB to serotonin transporters in conscious monkey brain. Synapse.

[29] Pehrson AL (2013). Lu AA21004, a novel multimodal antidepressant, produces regionally selective increases of multiple neurotransmitters-A rat microdialysis and electrophysiology study. Eur. Neuropsychopharmacol..

[30] Jayaram-Lindström N (2017). Naltrexone modulates dopamine release following chronic, but not acute amphetamine administration: a translational study. Transl. Psychiatry.

[31] Oswald LM (2015). Risky decision-making and ventral striatal dopamine responses to amphetamine: a positron emission tomography [11C]raclopride study in healthy adults. NeuroImage.

[32] Volkow ND (2015). Recovery of dopamine transporters with methamphetamine detoxification is not linked to changes in dopamine release. Neuroimage.

[33] Lanteri C (2014). Repeated exposure to MDMA triggers long-term plasticity of noradrenergic and serotonergic neurons. Mol. Psychiatry.

[34] Baumann MH, Williams Z, Zolkowska D, Rothman RB (2011). Serotonin (5-HT) precursor loading with 5-hydroxy-l-tryptophan (5-HTP) reduces locomotor activation produced by (+)-amphetamine in the rat. Drug Alcohol Depend..

[35] National Research Council. *Guide for the Care and Use of Laboratory Animals* (The National Academies Press, Washington, DC, 2011).

[36] Andersson JD (2011). Development of a PET radioligand for the central 5-HT1B receptor: radiosynthesis and characterization in cynomolgus monkeys of eight radiolabeled compounds. Nucl. Med. Biol..

[37] Varrone A (2009). Advancement in PET quantification using 3D-OP-OSEM point spread function reconstruction with the HRRT. Eur. J. Nucl. Med. Mol. Imaging.

[38] Tustison NJ (2010). N4ITK: improved N3 bias correction. IEEE Trans. Med. Imaging.

[39] Avants BB (2010). The optimal template effect in hippocampus studies of diseased populations. Neuroimage.

[40] Rohlfing T (2012). The INIA19 template and NeuroMaps atlas for primate brain image parcellation and spatial normalization. Front. Neuroinformatics.

[41] Paxinos, G., Huang, X.-F., Petrides M. & Toga A. *The Rhesus Monkey Brain in Stereotaxic Coordinates* (Academic Press, San Diego, 2008).

[42] Calabrese E (2015). A diffusion tensor MRI atlas of the postmortem rhesus macaque brain. Neuroimage.

[43] Avants BB, Epstein CL, Grossman M, Gee JC (2008). Symmetric diffeomorphic image registration with cross-correlation: evaluating automated labeling of elderly and neurodegenerative brain. Med. Image Anal..

[44] Nord M, Finnema SJ, Schain M, Halldin C, Farde L (2014). Test-retest reliability of [11C]AZ10419369 binding to 5-HT1B receptors in human brain. Eur. J. Nucl. Med. Mol. Imaging.

[45] Varnäs K (2011). Quantitative analysis of [11C]AZ10419369 binding to 5-HT1B receptors in human brain. J. Cereb. Blood Flow Metab..

[46] Fleckenstein AE, Volz TJ, Riddle EL, Gibb JW, Hanson GR (2007). New insights into the mechanism of action of amphetamines. Annu. Rev. Pharmacol. Toxicol..

[47] Heal DJ, Smith SL, Gosden J, Nutt DJ (2013). Amphetamine, past and present--a pharmacological and clinical perspective. J. Psychopharmacol..

[48] Narendran R (2004). In vivo vulnerability to competition by endogenous dopamine: comparison of the D2 receptor agonist radiotracer (-)-N-[11C]propyl-norapomorphine ([11C]NPA) with the D2 receptor antagonist radiotracer [11C]-raclopride. Synapse.

[49] Seneca N (2006). Effect of amphetamine on dopamine D2 receptor binding in nonhuman primate brain: a comparison of the agonist radioligand [11C]MNPA and antagonist [11C]raclopride. Synapse.

[50] Martinez D (2003). Imaging human mesolimbic dopamine transmission with positron emission tomography. Part II: amphetamine-induced dopamine release in the functional subdivisions of the striatum. J. Cereb. Blood Flow Metab..

[51] Erritzoe D (2017). Serotonin release measured in the human brain: A PET study with [C-11] Cimbi-36 and damphetamine challenge. J. Cereb. Blood Flow Metab..

[52] Aalto S (2009). The effects of d-amphetamine on extrastriatal dopamine D2/D 3 receptors: a randomized, double-blind, placebo-controlled PET study with [11C]FLB 457 in healthy subjects. Eur. J. Nucl. Med. Mol. Imaging.

[53] Kehr J (2011). Mephedrone, compared with MDMA (ecstasy) and amphetamine, rapidly increases both dopamine and 5-HT levels in nucleus accumbens of awake rats. Br. J. Pharmacol..

[54] Bowyer JF (2003). Plasma levels of parent compound and metabolites after doses of either d-fenfluramine or d-3,4-methylenedioxymethamphetamine (MDMA) that produce long-term serotonergic alterations. Neurotoxicology.

[55] Gamma A, Buck A, Berthold T, Hell D, Vollenweider FX (2000). 3,4-Methylenedioxymethamphetamine (MDMA) modulates cortical and limbic brain activity as measured by [H(2)(15)O]-PET in healthy humans. Neuropsychopharmacology.

[56] Tyacke RJ, Nutt DJ (2015). Optimising PET approaches to measuring 5-HT release in human brain. Synapse.

[57] Vizeli P, Liechti ME (2017). Safety pharmacology of acute MDMA administration in healthy subjects. J. Psychopharmacol..

[58] Jacobsen JP (2016). SSRI augmentation by 5-hydroxytryptophan slow release: mouse pharmacodynamic proof of concept. Neuropsychopharmacology.

[59] Visser AKD (2011). Measuring serotonin synthesis: from conventional methods to PET tracers and their (pre)clinical implications. Eur. J. Nucl. Med. Mol. Imaging.

[60] Frick A (2015). Serotonin synthesis and reuptake in social anxiety disorder: a positron emission tomography study. JAMA Psychiatry.

[61] Agren H (1991). Low brain uptake of L-[11C]5-hydroxytryptophan in major depression: a positron emission tomography study on patients and healthy volunteers. Acta Psychiatr. Scand..

[62] Hagberg GE (2002). Kinetic compartment modeling of [11C]-5-hydroxy-L-tryptophan for positron emission tomography assessment of serotonin synthesis in human brain. J. Cereb. Blood Flow Metab..

[63] Lundquist P (2006). Validation studies on the 5-hydroxy-L-[β-11C]-tryptophan/ PET method for probing the decarboxylase step in serotonin synthesis. Synapse.

[64] Finnema SJ (2015). Amphetamine decreases α2C-adrenoceptor binding of [11C]ORM-13070: a PET study in the primate brain. Int. J. Neuropsychopharmacol..

[65] Pum M, Carey RJ, Huston JP, Müller CP (2007). Dissociating effects of cocaine and d-amphetamine on dopamine and serotonin in the perirhinal, entorhinal, and prefrontal cortex of freely moving rats. Psychopharmacology.

[66] Nair AB, Jacob S (2016). A simple practice guide for dose conversion between animals and human. J. Basic Clin. Pharm..

[67] US Food and Drug Administration. *Guidance for Industry: Estimating the Maximum Safe Starting Dose in Initial Clinical Trials for Therapeutics in Adult Healthy Volunteers* (US Dept. of Health and Human Services, Food and Drug Administration, Center for Drug Evaluation and Research (CDER), Rockville, 2005).

[68] Mueller M, Goodwin AK, Ator NA, McCann UD, Ricaurte GA (2011). Metabolism and disposition of 3,4-methylenedioxymethamphetamine (“Ecstasy”) in baboons after oral administration: comparison with humans reveals marked differences. J. Pharmacol. Exp. Ther..

[69] Cook CE (1993). Pharmacokinetics of methamphetamine self-administered to human subjects by smoking S-(+)-methamphetamine hydrochloride. Drug Metab. Dispos..

[70] Magnussen I, Nielsen-Kudsk F (1980). Bioavailability and related pharmacokinetics in man of orally administered L-5-hydroxytryptophan in steady state. Acta Pharmacol. Toxicol..

[71] Westenberg HGM, Gerritsen TW, Meijer BA, van Praag HM (1982). Kinetics of l-5-hydroxytryptophan in healthy subjects. Psychiatry Res..

[72] Besnard J (2012). Automated design of ligands to polypharmacological profiles. Nature.

[73] Yamanaka H (2014). A possible mechanism of the nucleus accumbens and ventral pallidum 5-HT1B receptors underlying the antidepressant action of ketamine: a PET study with macaques. Transl. Psychiatry.

[74] Yamamoto S (2013). Subanesthetic doses of ketamine transiently decrease serotonin transporter activity: a PET study in conscious monkeys. Neuropsychopharmacology.

[75] Neumaier JF, Vincow ES, Arvanitogiannis A, Wise RA, Carlezon WA (2002). Elevated expression of 5-HT1B receptors in nucleus accumbens efferents sensitizes animals to cocaine. J. Neurosci..

[76] Varnäs K (2011). Dose-dependent binding of AZD3783 to brain 5-HT1B receptors in non-human primates and human subjects: a positron emission tomography study with [11C]AZ10419369. Psychopharmacology.

