# Supplementary material for: Serotonin concentration enhancers at clinically relevant doses reduce [11C]AZ10419369 binding to the 5-HT1B receptors in the nonhuman primate brain
Source: Transl Psychiatry. 2018 Jul 16;8:132. doi: 10.1038/s41398-018-0178-7 (PMC6048172; doi:10.1038/s41398-018-0178-7)
Supplement: Supplementary file 1 — Supplementary information_clear [file 41398_2018_178_MOESM1_ESM.docx]

**Supplementary information**

**Supplementary Methods**

**Sample size estimation**

The sample size estimation for the current study was performed using G*Power (version 3.1.9.2; <http://www.gpower.hhu.de/>)[^1^](#_ENREF_1). The effect size was calculated based on the reported effect of escitalopram on [^11^C]AZ10419369 binding in the raphe nucleus[^2^](#_ENREF_2) and the correlation between groups was set to 0.9. The parameters for sample size calculation were two-tails, effect size: 2.25, α= 0.05 and power = 0.50 (for the exploratory purpose of the current study). The estimated sample size was 3.

**Cynomolgus brain template**

Thirty-six healthy cynomolgus monkeys (*Macaca fascicularis*) were included for template creation (female/male: 28/8; age: 3.9 ± 0.6 years old and body weight: 3.7 ± 0.9 Kg). Several image preprocessing steps were applied. First, individual whole head MRI images were resampled to 0.5 mm isotropic voxel size and reoriented along the anterior commissure and posterior commissure (AC-PC) plane manually. Then, the MRI image matrix was reduced to 256 x 256 x 128 and corrected for inhomogeneous intensity by applying the N4 algorithm[^3^](#_ENREF_3) in the Advanced Normalization Tools (ANTs) software package (<http://stnava.github.io/ANTs/>), operated in the platform of NeuroDebian (ANTs version: 2.1.0-2~nd80+1)[^4^](#_ENREF_4). The non-brain tissues were removed manually using ITK-SNAP (version: 3.4.0; <http://www.itksnap.org/>)[^5^](#_ENREF_5) and individual brain MRI images were corrected for inhomogeneous intensity again by applying the N4 algorithm. Next, the corrected brain MRI images were segmented using “fast” algorithm in the FMRIB Software Library (FSL) v5.0[^6^](#_ENREF_6). The intensities of all voxels in the gray matter (GM) segments were averaged to determine the average voxel intensity of each GM segment. Finally, the corrected brain MRI images were scaled to the average voxel intensity in GM while set the average GM intensity as 100.

The preprocessed brain MRI images were used to create the cynomolgus brain template using symmetric group-wise normalization (SyGN) procedures[^7^](#_ENREF_7) provided by ANTs. SyGN is a powerful framework for producing optimal population-specific templates that are unbiased in both shape and appearance of individual brain images. In addition to creation of human brain template, the approach has been successfully applied to build up MRI template for baboons[^8^](#_ENREF_8).

**Maps of volumes of interest (VOI)**

The majority of VOIs were delineated based on the NeuroMaps atlas in the INIA19 template[^9^](#_ENREF_9) which is one of most detailed atlas for the monkey brain and has been successfully transformed into vervet, a species of monkey other than rhesus[^10^](#_ENREF_10). For the regions not optimized on the NeuroMaps atlas,^7^ such as hippocampus, thalamus and midbrain, the Paxinos' histology atlas[^11^](#_ENREF_11) in the CIVM template[^12^](#_ENREF_12) was applied. The CIVM template was firstly normalized to the INIA19 template by the antsRegistration algorithm using symmetric image normalization method (SyN)[^13^](#_ENREF_13) in ANTs. The resulting normalization matrix was used to transform VOIs for hippocampus, thalamus and midbrain into the space of the INIA19 template. Then the INIA19 template was normalized to the in-house cynomolgus brain template by SyN and all VOIs (except the whole brain) were then transformed from the space of the INIA19 template to that of the in-house cynomolgus brain template using the same normalization matrix.

**Supplementary Results**

**Table S1.** Mean values of [^11^C]AZ10419369 injection parameters during baseline and pretreatment conditions

|  |  | Amphetamine (*n* = 3) ^#^ | |  | MDMA (*n* = 3) | |  | 5-HTP (*n* = 3) | |
| --- | --- | --- | --- | --- | --- | --- | --- | --- | --- |
|  |  | Baseline | PreTx |  | Baseline | PreTx |  | Baseline | PreTx |
| Injected dose (MBq) |  | 163 | 163 |  | 127 | 127 |  | 149 | 153 |
| SRA (GBq/μmol) |  | 335 | 377 |  | 596 | 610 |  | 369 | 371 |
| Injected mass (μg) |  | 0.56 | 0.58 |  | 0.34 | 0.32 |  | 0.59 | 0.50 |

PreTx, pretreatment; SRA, specific radioactivity.

^#^A total of 14 PET measurements were performed in 3 NHPs (NHP1: 6, NHP2: 4 and NHP3: 4; half for baseline conditions and half for pretreatment conditions). For each NHP, the mean value of all related PET measurements was used to represent the value of the NHP.

No significant difference (*P* < 0.05, two-tailed, paired *t*-test) between baseline and pretreatment condition was observed.

**Table S2.** Effect of amphetamine on regional binding potential (*BP*_ND_) values

| NHP1 | Baseline1 | Baseline2 | Baseline3 | PreTx1 | PreTx2 | PreTx3 | ∆*BP*_ND_ (%)1 | ∆*BP*_ND_ (%)2 | ∆*BP*_ND_ (%)3 |
| --- | --- | --- | --- | --- | --- | --- | --- | --- | --- |
| Frontal cortex | 0.93 | 0.76 | 0.86 | 0.77 | 0.68 | 0.61 | -17 | -10 | -28 |
| Occipital cortex | 1.23 | 1.29 | 1.23 | 0.90 | 0.81 | 0.82 | -27 | -37 | -33 |
| Hippocampus | 0.75 | 0.77 | 0.88 | 0.64 | 0.59 | 0.57 | -15 | -24 | -35 |
| Caudate nucleus | 1.04 | 0.99 | 1.13 | 0.75 | 0.83 | 0.75 | -27 | -17 | -34 |
| Putamen | 1.12 | 1.07 | 1.18 | 0.77 | 0.92 | 0.82 | -31 | -14 | -31 |
| Ventral striatum | 1.45 | 1.19 | 1.60 | 1.14 | 1.07 | 1.03 | -22 | -10 | -36 |
| Globus pallidus | 2.00 | 2.06 | 2.36 | 1.32 | 1.65 | 1.45 | -34 | -20 | -39 |
| Thalamus | 1.07 | 1.06 | 1.07 | 0.69 | 0.79 | 0.62 | -36 | -25 | -42 |
| Midbrain | 1.28 | 1.40 | 1.45 | 0.95 | 1.05 | 0.88 | -26 | -25 | -39 |
| Whole brain | 0.86 | 0.78 | 0.83 | 0.68 | 0.63 | 0.60 | -20 | -19 | -27 |
| NHP2 | Baseline1 | Baseline2 |  | PreTx1 | PreTx2 |  | ∆*BP*_ND_ (%)1 | ∆*BP*_ND_ (%)2 |  |
| Frontal cortex | 0.94 | 1.09 |  | 0.84 | 0.74 |  | -11 | -31 |  |
| Occipital cortex | 1.79 | 1.80 |  | 1.38 | 1.18 |  | -23 | -35 |  |
| Hippocampus | 1.01 | 0.93 |  | 0.85 | 0.74 |  | -16 | -20 |  |
| Caudate nucleus | 0.82 | 1.05 |  | 0.74 | 0.74 |  | -10 | -29 |  |
| Putamen | 1.10 | 1.27 |  | 1.01 | 0.88 |  | -8 | -30 |  |
| Ventral striatum | 1.59 | 1.79 |  | 1.44 | 1.23 |  | -9 | -31 |  |
| Globus pallidus | 2.05 | 1.92 |  | 1.80 | 1.44 |  | -12 | -25 |  |
| Thalamus | 1.14 | 1.31 |  | 0.85 | 0.80 |  | -25 | -39 |  |
| Midbrain | 1.39 | 1.53 |  | 1.14 | 1.11 |  | -18 | -28 |  |
| Whole brain | 0.91 | 1.02 |  | 0.76 | 0.69 |  | -16 | -33 |  |
| NHP3 | Baseline1 | Baseline2 |  | PreTx1 | PreTx2 |  | ∆*BP*_ND_ (%)1 | ∆*BP*_ND_ (%)2 |  |
| Frontal cortex | 0.71 | 0.71 |  | 0.52 | 0.65 |  | -27 | -8 |  |
| Occipital cortex | 1.26 | 1.22 |  | 0.80 | 1.01 |  | -37 | -18 |  |
| Hippocampus | 0.91 | 0.83 |  | 0.60 | 0.82 |  | -33 | -1 |  |
| Caudate nucleus | 0.87 | 0.81 |  | 0.53 | 0.73 |  | -39 | -10 |  |
| Putamen | 0.97 | 0.91 |  | 0.61 | 0.91 |  | -37 | -1 |  |
| Ventral striatum | 1.61 | 1.25 |  | 1.17 | 1.32 |  | -27 | 6 |  |
| Globus pallidus | 1.72 | 1.60 |  | 1.18 | 1.19 |  | -32 | -26 |  |
| Thalamus | 1.01 | 0.81 |  | 0.64 | 0.72 |  | -36 | -11 |  |
| Midbrain | 1.27 | 0.96 |  | 0.89 | 0.97 |  | -30 | 1 |  |
| Whole brain | 0.72 | 0.69 |  | 0.48 | 0.63 |  | -33 | -9 |  |

PreTx: Pretreatment; ∆*BP*_ND_ (%) = $\frac{(\text{BP}_{\text{ND}}\text{Pretreatment} - \text{BP}_{\text{ND}}\text{Baseline})}{\text{BP}_{\text{ND}}\text{Baseline}}\text{×100}$.

**Supplementary References**

1. Faul F, Erdfelder E, Lang A-G, Buchner A. G*Power 3: A flexible statistical power analysis program for the social, behavioral, and biomedical sciences. *Behavior Research Methods* 2007; **39**(2)**:** 175-191.

2. Nord M, Finnema SJ, Halldin C, Farde L. Effect of a single dose of escitalopram on serotonin concentration in the non-human and human primate brain. *International Journal of Neuropsychopharmacology* 2013; **16:** 1577-1586.

3. Tustison NJ, Avants BB, Cook PA, Zheng Y, Egan A, Yushkevich PA*, et al*. N4ITK: Improved N3 bias correction. *IEEE Transactions on Medical Imaging* 2010; **29:** 1310-1320.

4. Halchenko YO, Hanke M. Open is not enough. Let's take the next step: An integrated, community-driven computing platform for neuroscience. *Frontiers in Neuroinformatics* 2012; **6:** 22.

5. Yushkevich PA, Piven J, Hazlett HC, Smith RG, Ho S, Gee JC*, et al*. User-guided 3D active contour segmentation of anatomical structures: Significantly improved efficiency and reliability. *NeuroImage* 2006; **31:** 1116-1128.

6. Jenkinson M, Beckmann CF, Behrens TEJ, Woolrich MW, Smith SM (2012). FSL. In: *NeuroImage*. pp 782-790.

7. Avants BB, Yushkevich P, Pluta J, Minkoff D, Korczykowski M, Detre J*, et al*. The optimal template effect in hippocampus studies of diseased populations. *NeuroImage* 2010; **49:** 2457-2466.

8. Love SA, Marie D, Roth M, Lacoste R, Nazarian B, Bertello A*, et al*. The average baboon brain: MRI templates and tissue probability maps from 89 individuals. *NeuroImage* 2016; **132:** 526-533.

9. Rohlfing T, Kroenke CD, Sullivan EV, Dubach MF, Bowden DM, Grant Ka*, et al*. The INIA19 template and NeuroMaps atlas for primate brain image parcellation and spatial normalization. *Frontiers in Neuroinformatics* 2012; **6:** 27.

10. Maldjian JA, Daunais JB, Friedman DP, Whitlow CT. Vervet MRI atlas and label map for fully automated morphometric analyses. *Neuroinformatics* 2014; **12:** 543-550.

11. Paxinos G, Huang X-F, Michael P, Toga A. The Rhesus Monkey Brain in Stereotaxic Coordinates. 2008.

12. Calabrese E, Badea A, Coe CL, Lubach GR, Shi Y, Styner MA*, et al*. A diffusion tensor MRI atlas of the postmortem rhesus macaque brain. *NeuroImage* 2015; **117:** 408-416.

13. Avants BB, Epstein CL, Grossman M, Gee JC. Symmetric diffeomorphic image registration with cross-correlation: Evaluating automated labeling of elderly and neurodegenerative brain. *Medical Image Analysis* 2008; **12:** 26-41.
